# Manipulating light scattering and optical confinement in vertically stacked Mie resonators

**DOI:** 10.1515/nanoph-2022-0605

**Published:** 2022-11-11

**Authors:** Felix Vennberg, Ajith Padyana Ravishankar, Srinivasan Anand

**Affiliations:** Applied Physics, KTH Royal Institute of Technology School of Engineering Sciences, Hannes Alféns väg 12, 114 19, Stockholm, Sweden

**Keywords:** anapole, colloidal lithography, III–V nanodisks, Mie resonances, optical confinement, vertical stack

## Abstract

High index dielectric nanoresonators have gained prominence in nanophotonics due to lower losses compared to plasmonic systems and their ability to sustain both electric and magnetic resonances. The resonances can be engineered to create new types of optical states, such as bound-states in a continuum (BIC) and anapoles. In this work, we report on the optical properties of vertically stacked AlGaAs nanodisk Mie resonators. The nanodisks are designed to support an anapole state in the visible wavelength region (400–700 nm). The vertically stacked nanodisk resonators are fabricated from AlGaAs/GaAs multilayer samples with a fast and scalable patterning method using charged sphere colloidal lithography. Both measurements and finite difference time domain (FDTD) simulations of two and three stacked resonators show a sharp dip in the reflectance spectra at the anapole wavelength. For the 2 and 3 disk stacks the reflectance dip contrast at the anapole wavelength becomes very pronounced in the specular reflectance and is attributed to increased directional scattering due to an antenna effect. FDTD simulations show there is enhanced field confinement in all the disks at the anapole wavelength and the confined energy within the individual disks in the stack is at least 2–5 times greater compared to an isolated single nanodisk of the same dimension. Furthermore, the field confinement consistently increases with adding more disks in the stack. These vertically stacked AlGaAs nanodisk resonators can be a very exciting platform to engineer light matter interactions for linear and non-linear optical applications. The general principles of the fabrication method can be adapted to other wavelength ranges and can also be adapted for other III–V material combinations as well as for Si/SiO_2_.

## 1 Introduction

For over a decade, all-dielectric nanostructures have been studied to engineer electromagnetic interactions at nanoscales [1]. The focus has been on achieving optical resonances that can be utilized for applications such as structural color [2, 3], boosting nonlinear effects [4], filters [5], flat lenses and metamaterials [6, 7]. Dielectric structures with sizes comparable to the wavelength support Mie resonances. The scattering cross section (SCS) of these resonators can be characterized from the multipole sources induced within the resonators due to an external light source [8]. Multipole decomposition (MPD) of resonators with arbitrary geometries was recently developed, where the SCS can be calculated exactly from the electric charge-current distribution inside the resonator [9]. In contrast to plasmonic structures, Mie resonances in all-dielectric resonators can be both electric and magnetic. The interference between such resonances inside the resonator enables engineering of the SCS and manipulation of the near fields. For example, concepts such as bound states in the continuum [10, 11], fano resonances [12] and anapoles [13], have been realized using Mie resonance engineering. The anapole is a radiation-less state arising due to the interaction of one of the multipoles and its toroidal counterpart with the same far field radiation pattern. If the multipole and its toroidal component are completely out of phase they interfere destructively, so that no radiation escapes to the far field at that wavelength [14]. All multipoles can form an anapole state under certain conditions as shown with the recently discovered hybrid anapole (HA) state [15, 16]. However, the electric dipole anapole (EDA) is most commonly studied, possibly since it is relatively easier to realize. In this paper, we address the EDA interaction between nanodisks, and for simplicity we use the term anapole to refer to the EDA. However, observation of the anapole state requires higher order multipoles to be suppressed or they will dominate over the anapole signal (dip). Suppression of such interfering higher order multipoles can be achieved with low aspect ratio structures such as disks [13], or by using HA [15, 16]. The anapole state is characterized by a sharp decrease in the SCS and a specific localized field distribution with high confinement. These unique properties of the field confinement makes dielectric nanodisks supporting anapoles suitable candidates to enhance nonlinear processes such as harmonic generation [17, 18], frequency conversion [19], Kerr effect [20] and in general increase light matter interaction. Anapole states in Mie resonant dielectrics were first observed in Si nanodisks [13], and subsequently in III–V compounds [21, 22]. Other than their high refractive indices (RI), advantages of the III–Vs include their electronic band structure, a wider range of refractive indices, and higher nonlinear coefficients (e.g. *χ* ^(2)^) that are important in opto-electronic applications.

Placing resonators spatially close to each other can lead to interesting near-field interactions. For example, coherent near-field interaction between ‘bright’-mode and ‘dark’-mode supporting nanoresonators can produce a narrow electromagnetically induced transparency-like resonance [23]. In another study, the third harmonic response from silicon nanodisk trimers was controlled by changing the distances between the resonators [24]. In the case of anapoles, that are highly localized and non-radiative, they do interact and leak energy in the near field. It is thus possible to propagate an anapole excitation through a chain of resonators in the lateral plane [25–27]. Another approach to couple resonators at subwavelength distances is by stacking them vertically. A recent study showed low loss MHz wireless power transfer using anapole resonant disks in the vertical domain, with a split ring metallic wire as source [28]. A similar mechanism for radiation transfer can be expected for vertically stacked anapole resonators in the optical wavelength region. However, these resonators need to be in the nanoscale.

It is possible to fabricate stacked resonators using multilayer structures with alternative layers having different materials, e.g. Si/SiO_2_ or AlGaAs/GaAs. There are several advantages in the adoption of a vertically stacked resonator design. Since the thickness of each layer can be controlled in the deposition process, the thickness of the individual resonators as well as the spacer layers can be tuned independently. This together with control over lateral size enables control of the individual Mie resonances inside the resonators and how they interact with each other in the vertical domain. Such interaction between individual resonators supporting the same type of anapole state (e.g. EDA) is not possible in single cylinder geometries such as the one for the HA system [15]. Further, by adding more layers one can increase the density of Mie scatterers. Importantly, from the fabrication point of view, such structures are highly scalable using processes such as colloidal or nanoimprint lithography [29]. In spite of the technological advantages, only a few works have employed the vertical stack for light manipulation functions. Recently, it has been shown that SHG efficiency from stacked AlGaAs resonators can be enhanced by one order of magnitude and GaAs multilayer resonators can achieve ∼100% reflectance in near-infrared wavelength range, exceeding the reflectance from gold in the same wavelength range [30, 31]. Further, simulation results have shown that it is possible to attain strong coupling between stacked Si nanodisk and realize hybridized modes [32, 33].

In this work, we experimentally demonstrate the presence of anapole states in stacked Al_0.65_Ga_0.35_As nanodisks, and show how the characteristics of the anapole state are affected by the number of disks in a stack. To the best of our knowledge, vertically stacked AlGaAs resonators with anapole resonances have not been realized before. For the fabrication of stacked resonators, AlGaAs/GaAs is an ideal material system due to the well-established epitaxial growth techniques, dry etching recipes and availability of material selective wet etchants. It is possible to etch AlGaAs/GaAs in the same dry etch chemistry and subsequently wet etch GaAs with high selectivity. The result is a vertical stack of AlGaAs disks separated by thin GaAs stems. The choice of Al_0.65_Ga_0.35_As is due to its high RI, indirect bandgap and transparency above the wavelength ∼540 nm, whereas GaAs with its direct bandgap at ∼ 870 nm absorbs in the visible region. Previous works that have addressed vertical AlGaAs Mie resonant structures have used selective oxidation to separate the resonators [30]. However, the lower refractive index contrast between the resonator and the spacer layers (AlOx) would lead to more leakage into the oxide and reduce the energy confined within the disks. The refractive index contrast can also be achieved by using epsilon near zero materials as spacer layers [18]. In our case, to maximize the index contrast and field confinement we choose to under etch the spacer layer (GaAs). To demonstrate the scalability of the lithography process, the structures have been fabricated on cm^2^ sized substrates. Finite difference time domain (FDTD) simulations show high field localization of the anapole states in each of the stacked nanodisks as well as their interaction. The energy localization (at the anapole wavelength) in an individual stacked resonator is 2–5 times greater than for a single isolated resonator of the same dimensions.

## 2 Optimization of disk dimensions

First, the dimensions of the stacked nanodisk resonators are optimized to support the anapole state within the nanodisk. The FDTD method is used to investigate excitation and near-field interactions of anapole states in single and multi-stacked nanodisk resonators. In this work, all the FDTD simulations were performed using commercial software – Lumerical [34]. Total field scattered field (TFSF) source, with PML boundary condition in *x*, *y*, *z* directions, is used to calculate scattering total cross-section (SCS) from single and stacked nanodisks. Field monitors are used to obtain induced electric field inside nanodisks. Multipole decomposition method described in the work by Alee et al. [9], was implemented to evaluate contributions of multipoles in the total scattering cross-section from a single nanodisk. RI data for Al_0.7_Ga_0.3_As (closest match to Al_0.65_Ga_0.35_As) is taken from the work by Aspens et al. [35, 36], and for GaAs from Palik [37]. The dimensions of the nanodisks (diameter∼100–500 nm, height 50 nm) were chosen such that the anapole state is excited in the visible wavelength ranges (400–700 nm).

**Figure 1:**
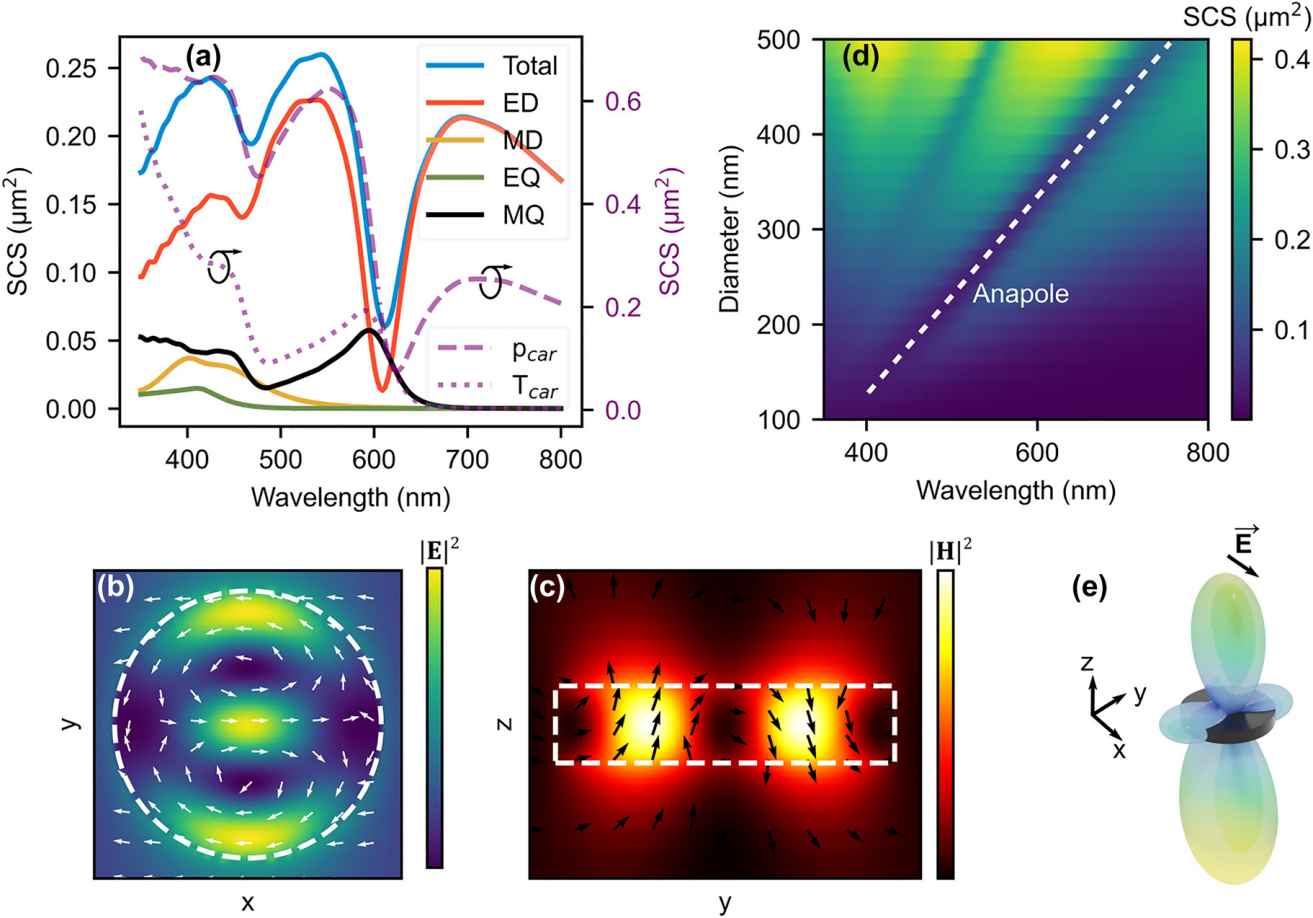
Scattering properties of a single AlGaAs disk in air. (a) Simulated scattering cross section (SCS) of a single AlGaAs nanodisk with diameter of 350 nm and thickness of 50 nm, placed in air (RI = 1). The figure also shows calculated multipole decomposition of the total SCS. The Cartesian toroidal (*T*_car_) and dipole (*p*_car_) contributions are shown in the same figure, with scale on the right side. The sharp dip (near 600 nm) in the electric dipole scattering corresponds to the excitation of the first order anapole. (b) ${\left|E\right|}^{2}$ at the center height of the disk, wavelength 610 nm. Arrows indicate displacement current direction (not scaled to magnitude) and the white dashed line is the disk border. (c) Magnetic field profile and flux direction. (d) The SCS’s dependence on disk diameter, the anapole state excitation wavelength redshifts with increasing diameter. (e) The far field radiation pattern close to anapole wavelength is dominated by the magnetic quadrupole. The arrow of the electric field indicates the polarization of the incoming light.

Figure 1(a) shows the simulated SCS of a single AlGaAs nanodisk, with a diameter of 350 nm and thickness of 50 nm, placed in air (RI = 1). The sharp dip near wavelength 610 nm is a typical signature of the anapole state. Multipole decomposition of the total SCS is also shown in Figure 1(a), where it is clear that the contribution of electric dipole (ED) to the total SCS approaches zero at the dip position, as expected for an anapole. It is also evident that due to the specific aspect ratio of the nanodisk, the scattering by the magnetic dipole is fully suppressed in the given wavelength range. However, the magnetic quadrupole (MQ) contributes to SCS at the anapole wavelength due to two antiparallel magnetic moments, seen in Figure 1(c). The magnetic quadrapole scattering is in fact a result of the displacement currents attributed to the anapole state in a disk [13]. Figure 1(b) and (c) shows the distribution of electric and magnetic fields inside the nanodisk, along with the field currents, that reveals the presence of electric and toroidal dipole sources. These are the localized field profiles typical to anapole excitation.

Figure 1(d) demonstrates the tunability of the anapole excitation wavelength in the visible region (350–800 nm). The diameter of the AlGaAs nanodisk is swept from 100 nm to 500 nm with a fixed nanodisk thickness of 50 nm, giving a linear shift in anapole excitation wavelength from 400 nm to 750 nm. The far field radiation at the anapole wavelength, Figure 1(e), has the expected quadrupole pattern. The far field pattern is discussed further in a later part of the paper.

**Figure 2:**
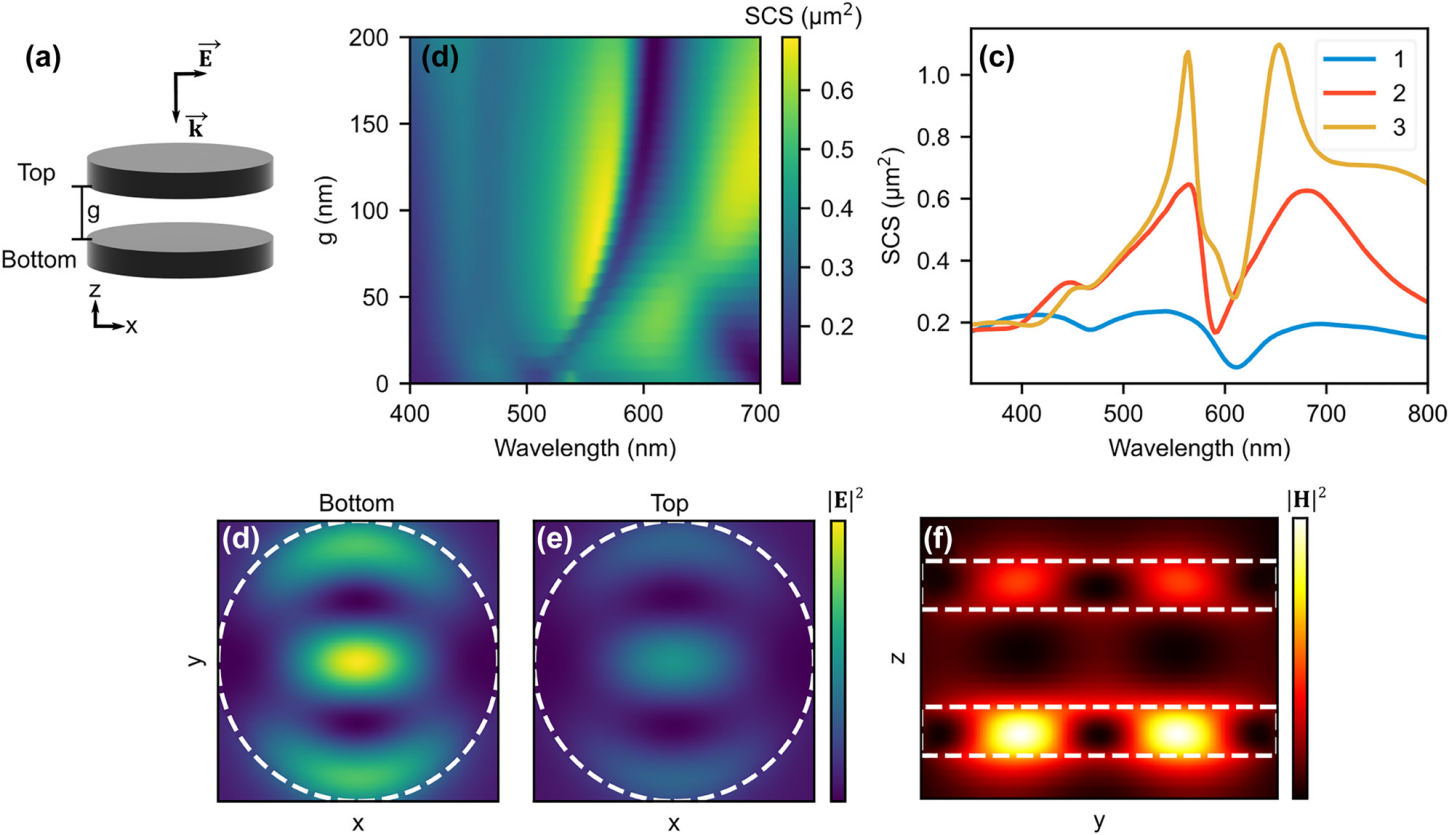
Scattering properties of two and three AlGaAs disks in air. (a) Schematic of two vertically stacked AlGaAs resonator separated by a gap distance of ‘*g*’. The incoming light is polarized in the *x* direction and traveling in the −*z* direction, as indicated by the $\vec{E}$ and $\vec{k}$ vectors. (b) Effect of changing the spatial gap between the two resonators on total scattering cross-section. Lowering the gap below 100 nm blue shifts the anapole dip and increasing the gap moves the dip towards wavelength position where anapole is excited for individual nanodisk. (c) Simulated SCS for one, two and three disk structures. The gap between the disks is 100 nm in the case of two and three stacked resonators. (d), and (e) Cross-sectional (XY) profiles of electric field intensity for respective disk. (f) Cross-sectional (YZ) profile of magnetic field (590 nm) shows that the anapole is excited in both the top and the bottom disks.

Next, we discuss how vertical stacking of AlGaAs nanodisks affects the total SCS and the anapole excitation wavelength. We begin by placing two disks in air separated by a gap distance ‘*g*’, see Figure 2(a). The disks are identical with a height of 50 nm and diameter 350 nm. The gap parameter was swept from 0 to 200 nm; the result is seen in Figure 2(b). For gaps up to 100 nm there is a dip with anapole like characteristics and its wavelength position red shifts with increasing gap. Above a gap of 100 nm it moves closer to that of an isolated single disk, as in Figure 1. The shift in dip wavelength as the disks move closer can be attributed to an increased near-field interaction between the two disks. Figure 2(c) compares the SCS of the double stack (*g* = 100 nm) to the SCS of a single disk. There is a clear increase in total SCS for the double stack, especially around the anapole wavelength position. We also show the effect of adding a third disk to the stack (*g* = 100 nm) on the SCS. The third disk continues the trend of the double stack and further increases the height of the peaks surrounding the anapole wavelength dip. A 2D map of the SCS for three disks is similar to the case with two nanodisks; the anapole dip position does not shift for ‘*g*’ values above 70 nm (see Figure SI 1).

The trend in the SCS for stacks up to three disks is a pronounced increase in contrast between the surrounding resonances and the anapole. The individual ED moments of the stacked disks are amplified around the anapole resonance, giving a larger contrast between the peaks and dips in the SCS. Figure 2(d) and (e) shows cross-sectional (XY plane) electric field profile of top and bottom disks at wavelength 590 nm. Similarly, Figure 2(f) shows cross-sectional (YZ) magnetic field profile of stacked nanodisks indicating field hot spots typical of an anapole. It is evident from the field profiles that the anapole is excited in both disks at the given wavelength. However, the electric and magnetic field intensity levels differ between the two disks. The bottom disk has stronger localized magnetic and electric fields.

**Figure 3:**
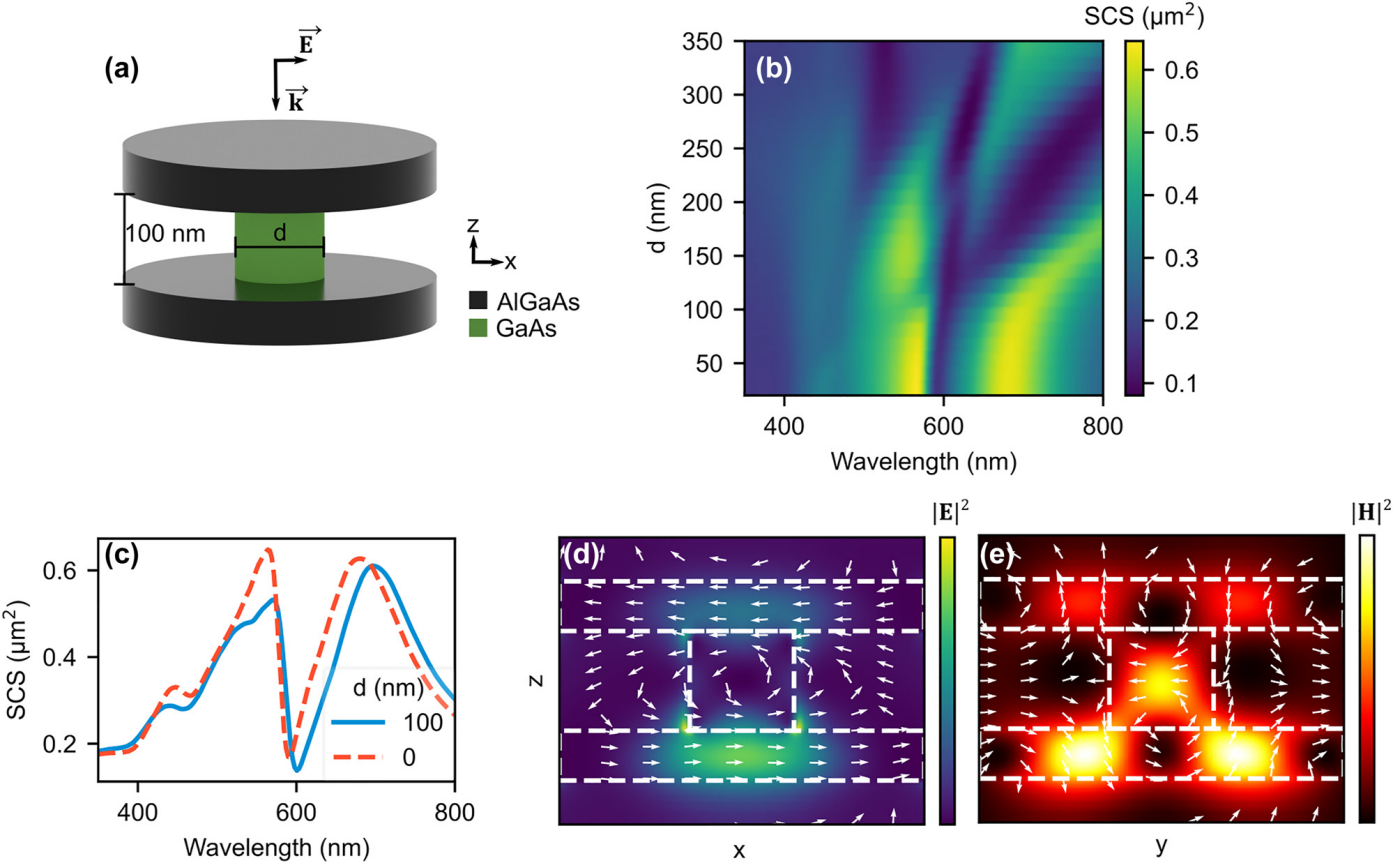
Influence of GaAs stem diameter on the scattering of two AlGaAs disks in air. (a) Schematic of two vertically stacked AlGaAs resonators connected with a GaAs stem with diameter ‘*d*’. The gap is kept at 100 nm. $\vec{E}$ and $\vec{k}$ vectors indicate polarization and direction of the incoming radiation, respectively. (b) Effect on the SCS as *d* is varied from 20 to 350 nm. (c) The total SCS of a two disk system connected via a 100 nm diameter GaAs stem (*d* = 100 nm), compared to the stem less system (*d* = 0). The magnitude squared of the electric (d) and magnetic (e) fields in the XZ plane at *y* = 0. The white arrows indicate respective field direction.

In reality, stacked resonators require support structures. We consider a system with a GaAs stem connecting the center of each disk, see Figure 3(a), and investigate what effect the GaAs stem diameter, ‘*d*’, has on the SCS. GaAs has similar refractive index to AlGaAs but is a direct bandgap semiconductor (*λ*_gap_ ∼ 870 nm) and absorbs light with wavelengths below 870 nm. The AlGaAs disks are kept at a diameter of 350 nm and a gap of 100 nm (as in the fabricated structures). From the SCS map in Figure 3(b), we see that for stem diameters below 100 nm, the SCS is almost invariant. When compared to the stemless system, see Figure 3(c), the *d* = 100 nm system maintains the overall shape of the SCS, but with a 10 nm redshift in the wavelength of the dip position. Furthermore, the anapole field patterns in the XY plane of each disk are retained, as seen in the supplementary information (Figure SI 2). Thus, a sufficiently narrow, high index stem connecting the disks does not destroy the anapole mode.

The electric and magnetic fields do however leak out to some extent, as seen in the YZ cross sections of Figure 3(d) and (e). The leakage and cross talk between the disks is greater for larger GaAs stem diameters. New resonances and possibly hybridized modes appear for stem diameters above 100 nm. Although new resonances and hybridized modes are interesting features, here we focus only on the anapole states, and restrict ourselves to GaAs stems with diameters of 100 nm or less.

## 3 Fabrication

**Figure 4:**
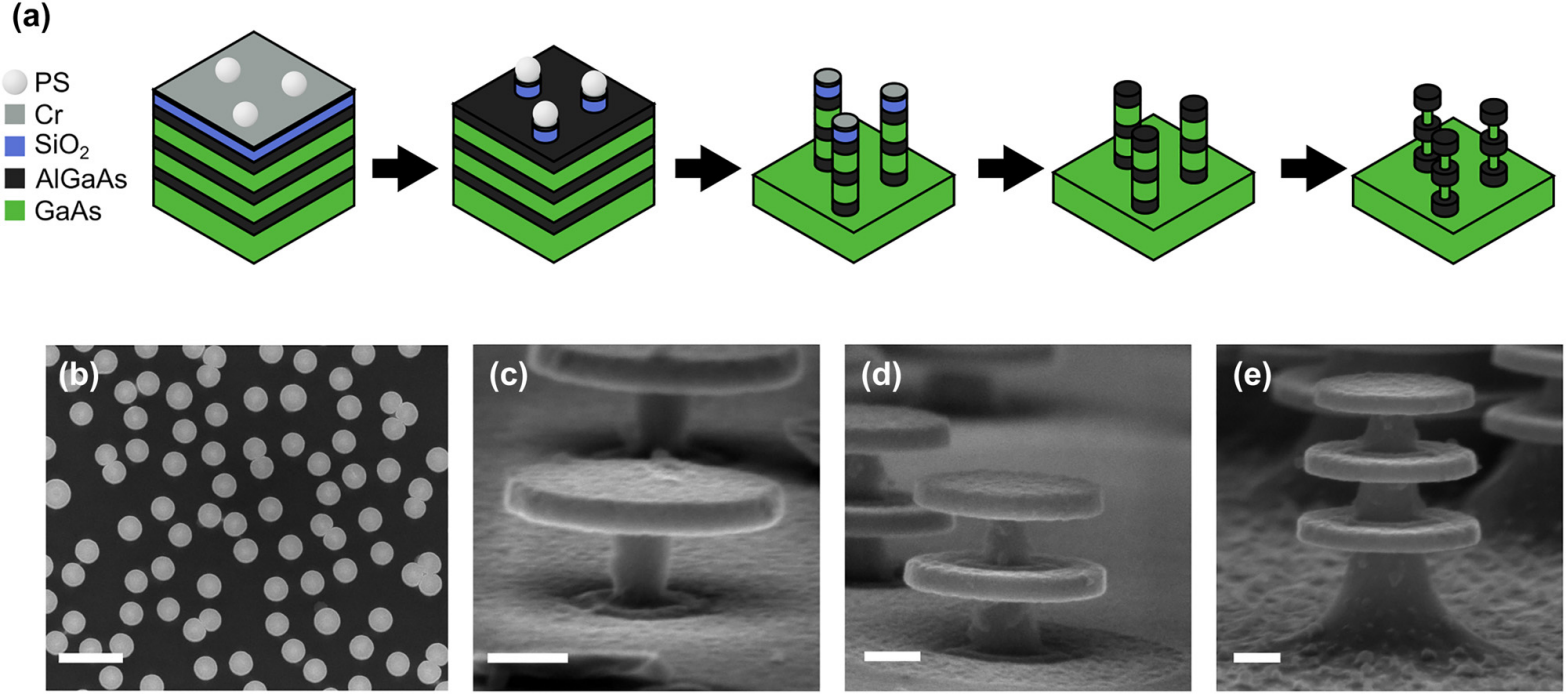
Fabrication steps to achieve stacked resonators. Start with AlGaAs/GaAs alternating multilayer sample, where the AlGaAs layers are 50 nm thick and the GaAs spacing layers are 100 nm thick. After deposition of Cr, SiO_2_, and charged polystyrene (PS) spheres, (a) the sphere pattern is transferred to the Cr and SiO_2_ through dry etching. The subsequent AlGaAs/GaAs dry etch removes the spheres and is timed to etch through either one, two or three of the AlGaAs layers. Both masks are removed in HF and the GaAs is wet etched, in a H_2_O_2_:citric acid mix. (b) A top view image of the final pillar structures, scale bar 1 µm. (c)–(e) Tilted views of the finalized one, two and three AlGaAs nanodisk stacks. The scale bars are 100 nm.

Following the results presented above, AlGaAs nanodisks with diameters of 350 nm connected by GaAs stems around 100 nm in diameter were fabricated. The AlGaAs/GaAs multilayer substrate used in this work consists of three layers of 50 nm thick Al_0.65_Ga_0.35_As separated by 100 nm thick GaAs layers, as shown in Figure 4(a). The top AlGaAs layer is capped with 20 nm GaAs to prevent oxidation. Processing steps, depicted in Figure 4(a), start with deposition of etch masks, SiO_2_ via plasma enhanced chemical vapor deposition (PECVD) followed by evaporation of Cr. After cleaning in acetone, IPA, and O_2_ plasma for 5 min, the sample is patterned through charged sphere colloidal lithography, a technique that can cover cm^2^ areas with nano sized polystyrene particles in minutes [38, 39]. The deposited particles form a mono-layer of randomly distributed particles with a nearest neighbor distance of about 1.5–2 diameters, although there is some bunching where particles are touching each other (Figure SI 3). Using the polystyrene particles as masks, the pattern is transferred into the Cr and subsequently into the SiO_2_, via dry etching processes in an inductively coupled plasma reactive ion etching (ICP-RIE). The Cr is etched with Cl_2_/O_2_ chemistry and the SiO_2_ with CHF_3_. The AlGaAs/GaAs multilayers are etched with a Cl_2_/Ar mixture in the ICP-RIE, and timed to stop after one, two or three AlGaAs layers are etched. The SiO_2_ mask, and the Cr with it, is removed in 5% buffered HF. The top view SEM image of the finalized structures in Figure 3(b) shows the lateral distribution of the etched pillars. The GaAs spacer layers are then selectively wet etched with 1:20 H_2_O_2_:citric acid mixture. The low amount of hydrogen peroxide gives a slow etch rate of 2 nm/s, which enables precise amounts of undercut. With this technique we can fabricate one, two or three disks in a vertical stack with a high degree of undercut of the GaAs layers, as seen in Figure 4(c)–(e).

## 4 Results & discussion

**Figure 5:**
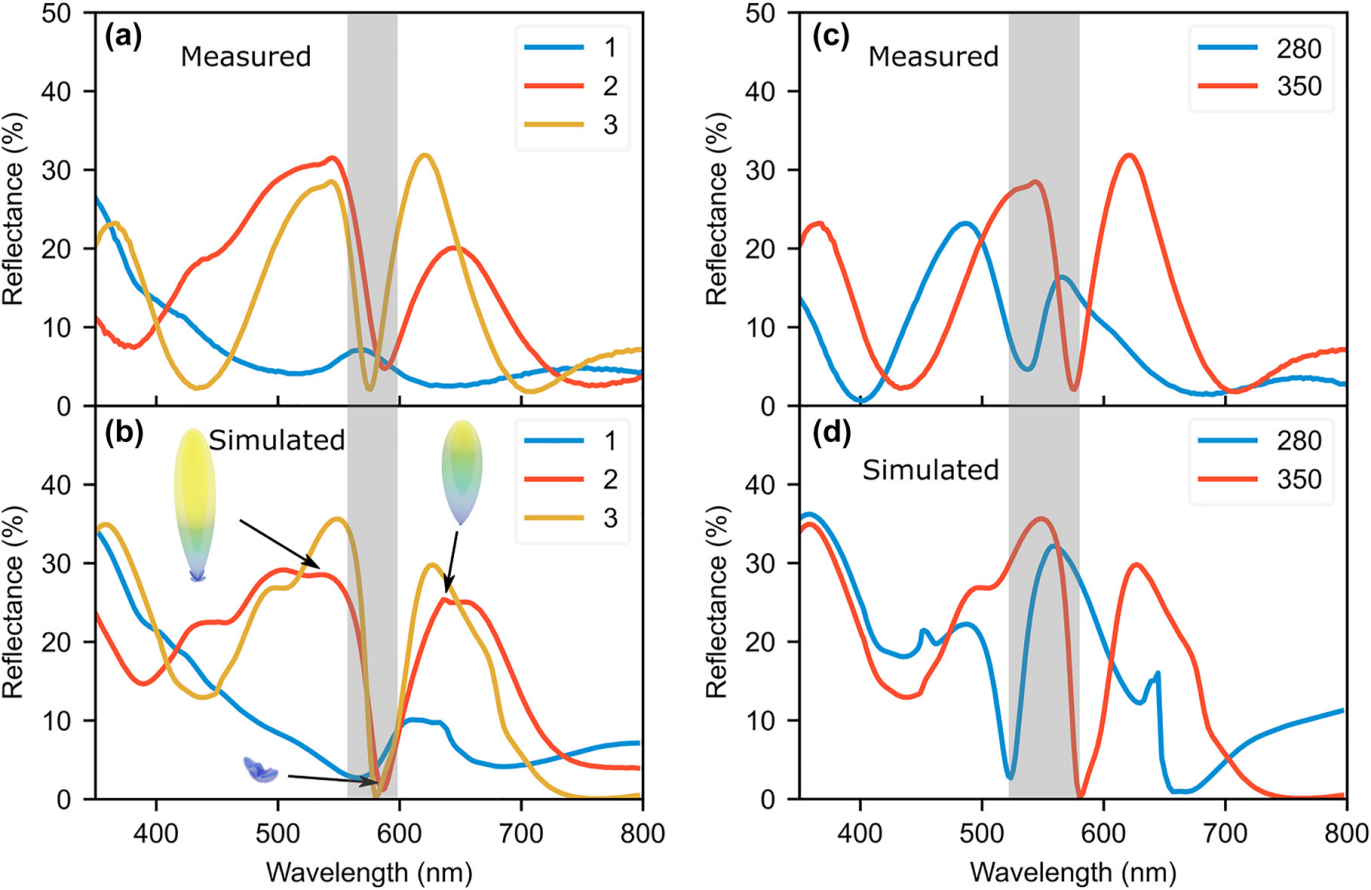
Specular reflectance of the disk stacks with AlGaAs diameter 350 nm and GaAs stem diameter 50 nm–100 nm. (a) Measured specular reflectance, the legend numbers indicate the number of disks in each stack. The corresponding simulated spectra are seen in (b), where the insets are the far field radiation plots for the reflected light in the two disk case. The far field plots are on the same scale, the size and color indicate magnitude of radiation. (c) Measured specular reflectance from three disk stacks with diameters 280 nm and 350 nm. (d) The corresponding simulated spectra.

The reflectances of the fabricated one, two and three stacked AlGaAs resonator samples were measured with Perkin Elmer lambda 900 spectrophotometer with an integrating sphere. The total and diffuse reflectance is measured independently and the specular reflectance is then calculated as the difference between the total and the diffuse reflectance. The anapole signature is most evident in the specular reflectance spectra, compared to the total reflectance spectra (see Figure SI 4). Hence, we focus our discussions on the measured specular reflectance data. The specular reflectance in the wavelength range 350–800 nm for disks with diameter 350 nm in Figure 5(a) agrees well with the SCS of Figure 3(c) and the simulated reflectance in Figure 5(b). The dip in the reflectance occurs at the expected anapole wavelength range (590–600 nm) for the stacks of two and three disks. The one disk case has a peak at the anapole wavelength due to the finite contribution of MQ resonance to the overall scattering, as seen in the MPD, Figure 1(a). Furthermore, the far field of one disk, Figure 1(e), has a lobe extending in the negative *z* direction, i.e., in the specular reflection.

The reflectance spectra of the 2 and 3 disk stacks were also replicated in simulations using a periodic array (Figure 5(b)) which agrees well with the measurements. The array period (900 nm) is such that the fill factor of the nanodisk matches the real samples, as calculated from top view SEM images (Figure 4(b)). The agreement of the simulation with experiment, even though the real structures are randomly distributed, suggests that the reflectance is a result of the individual resonances of the stacks and not from lattice effects. This is also corroborated by the fact that the SCS of a single disk structure matches well with the experimental reflectance data. The simulated stacks have stems with dimensions taken from the SEM images in Figure 4(c)–(e) and are on substrates with the appropriate number of layers underneath, i.e., the one disk has two layers of AlGaAs/GaAs underneath, and so on. From the simulations, we calculate the far field radiation for the reflected light in the two disk case and is shown in the insets of Figure 5(b). The far field for the ED resonances on either side of the anapole has large lobes in the vertical direction, i.e., strong directionality in the backscattered direction. The far field at the anapole wavelength is small in comparison. We also show the shift in anapole wavelength with diameter in Figure 5(c), where a stack of three disks with diameter 280 nm has an anapole wavelength at 540 nm, consistent with the map in Figure 1(b) and corresponding simulated reflectance in Figure 5(d).

**Figure 6:**
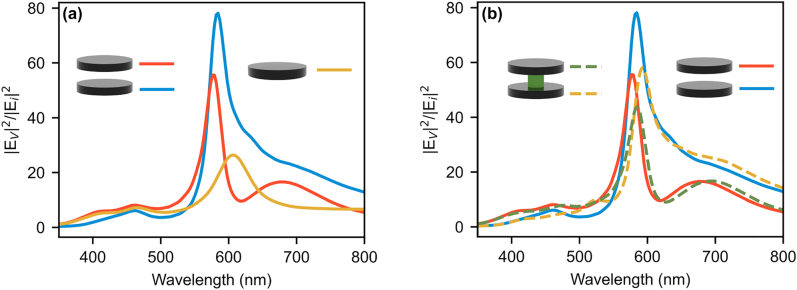
Energy inside the resonators ${\left|E_{v}\right|}^{2}$ normalized to the incoming energy ${\left|E_{i}\right|}^{2}$ . (a) Comparison of the energy inside each disk in the two disk case and the single disk. Simulations are in air with an air gap of 100 nm between the disks. (b) Comparison of internal energy inside the nanodisks of two-disk stack with 100 nm diameter GaAs stem (dashed lines) and without GaAs stem (solid lines).

To better understand the mechanism behind the increased contrast and the consequence on field confinement in the multi disk cases, we calculate the total energy ( ${\left|E_{v}\right|}^{2}$ ) inside each disk. As a measure of the localization of energy we normalize with the incoming energy, ${\left|E_{i}\right|}^{2}$ , giving the expression ${\left|E_{v}\right|}^{2}/{\left|E_{i}\right|}^{2}=\int_{V}\varepsilon_{r}{\left|E\right|}^{2}dV/\left({\left|E_{i}\right|}^{2}V\right)$ , where *ɛ*_*r*_ is the relative permittivity, *E* is the induced electric field inside the disk and *V* the disk volume. We have neglected the imaginary part of the permittivity since the Al_0.65_Ga_0.35_As is non-absorbing for wavelengths above ∼520 nm, where the features of interest are located. As seen in Figure 6(a), there is a characteristic peak in the energy at the anapole wavelength for both the single and two disk cases. The calculated energy inside each disk in the 2 and 3 disk stack cases is greater than that of the single disk. Stacking disks vertically not only gives an increased contrast due to enhanced ED outside of anapole, but also an increase in electric field localization. Furthermore, the bottom disk has higher confinement energy than the top one. These observations suggest that the anapole state leaks energy from the top to the bottom. Since the fabricated structures have a GaAs stem, the confined energy is also calculated for disk stacks with 100 nm diameter GaAs stems, and the results are shown on Figure 6(b). Although the confinement energies are lower compared to the disks in air, they are still higher than that for the single disk in air. The same analysis on a three-disk stack (SI 5a and b) shows that overall field confinement in the stack is ∼2–3 times higher compared to a two disk stack and an order of magnitude larger than the single disk resonator system. This trend of higher field confinement with more number of disks per stack is observed even for four and five-disk stacks (Figure SI 5d), especially at anapole wavelengths. With increasing number of disks in a stack, additional peaks are seen in the confinement energy spectra of the nanodisks that may be attributed to the excitation of other Mie modes. However, field confinement at anapole wavelength consistently increases with the addition of more disks in the vertical stack.

## 5 Conclusions

We have fabricated stacked AlGaAs nanodisk resonators using AlGaAs/GaAs multilayers and have demonstrated the excitation of anapole state in two and three stacked resonator systems in the wavelength range of 400–700 nm. The total and specular reflectance spectra from these structures were analysed using Mie scattering. For the 2 and 3 disk stacks the reflectance dip contrast at the anapole wavelength becomes very pronounced in the specular reflectance and is attributed to increased directional scattering. The anapole state is experimentally observable even when the disks are supported by thin GaAs stems at the center. We also show that it is possible to propagate the anapole state from resonator to resonator in the vertical direction and that the coupling between the disks can be tuned by changing the gap between the disks or by varying the diameter of the GaAs stem. Simulations show that the internal electric energy in each disks of the two and three stacked resonators is enhanced by 2–5 times as compared to an isolated single disk. The field enhancement increases with the addition of more disks to the vertical stack. We also show a method of fabricating stacked nanodisk resonators on a large scale based on charged sphere colloidal lithography and etching. These vertically stacked AlGaAs nanodisk resonators can be a very exciting platform to engineer light matter interactions for linear and non-linear optical applications. The general principles of the fabrication method can be adapted to other wavelength ranges and can also be adapted for other III–V material combinations as well as for Si/SiO_2_.

## Supplementary Material

Supplementary Material Details

## References

[1] Kuznetsov A. I., Miroshnichenko A. E., Brongersma M. L., Kivshar Y. S., Luk’yanchuk B. (2016). Optically resonant dielectric nanostructures. *Science*.

[2] Proust J., Bedu F., Gallas B., Ozerov I., Bonod N. (2016). All-dielectric colored metasurfaces with silicon Mie resonators. ACS Nano.

[3] Neder V., Luxembourg S. L., Polman A. (2017). Efficient colored silicon solar modules using integrated resonant dielectric nanoscatterers. Appl. Phys. Lett..

[4] Liu S., Sinclair M. B., Saravi S. (2016). Resonantly enhanced second-harmonic generation using III–V semiconductor all-dielectric metasurfaces. Nano Lett..

[5] Visser D., Basuvalingam S. B., Désières Y., Anand S. (2019). Optical properties and fabrication of dielectric metasurfaces based on amorphous silicon nanodisk arrays. Opt. Express.

[6] Jahani S., Jacob Z. (2016). All-dielectric metamaterials. Nat. Nanotechnol..

[7] Staude I., Schilling J. (2017). Metamaterial-inspired silicon nanophotonics. Nat. Photonics.

[8] Bohren C. F., Huffman D. R. (2008). *Absorption and Scattering of Light by Small Particles, 1. Aufl.*.

[9] Alaee R., Rockstuhl C., Fernandez-Corbaton I. (2018). An electromagnetic multipole expansion beyond the long-wavelength approximation. Opt. Commun..

[10] Koshelev K., Kruk S., Melik-Gaykazyan E. (2020). Subwavelength dielectric resonators for nonlinear nanophotonics. Science.

[11] Rybin M. V., Koshelev K. L., Sadrieva Z. F. (2017). High- Q supercavity modes in subwavelength dielectric resonators. Phys. Rev. Lett..

[12] Yang Y., Wang W., Boulesbaa A. (2015). Nonlinear fano-resonant dielectric metasurfaces. Nano Lett..

[13] Miroshnichenko A. E., Evlyukhin A. B., Yu Y. F. (2015). Nonradiating anapole modes in dielectric nanoparticles. Nat. Commun..

[14] Yang Y., Bozhevolnyi S. I. (2019). Nonradiating anapole states in nanophotonics: from fundamentals to applications. *Nanotechnology*.

[15] Canós Valero A., Gurvitz E. A., Benimetskiy F. A. (2021). Theory, observation, and ultrafast response of the hybrid anapole regime in light scattering. Laser Photonics Rev..

[16] Kuznetsov A. V., Valero A. C., Tarkhov M., Bobrovs V., Redka D., Shalin A. S. (2021). Transparent hybrid anapole metasurfaces with negligible electromagnetic coupling for phase engineering. Nanophotonics.

[17] Grinblat G., Li Y., Nielsen M. P., Oulton R. F., Maier S. A. (2016). Enhanced third harmonic generation in single germanium nanodisks excited at the anapole mode. Nano Lett..

[18] Li Y., Huang Z., Sui Z. (2020). Optical anapole mode in nanostructured lithium niobate for enhancing second harmonic generation. Nanophotonics.

[19] Gili V. F., Ghirardini L., Rocco D. (2018). Metal-dielectric hybrid nanoantennas for efficient frequency conversion at the anapole mode. Beilstein J. Nanotechnol..

[20] Panov A. V. (2020). Optical Kerr nonlinearity of arrays of all-dielectric high-index nanodisks in the vicinity of the anapole state. Opt. Lett..

[21] Timofeeva M., Lang L., Timpu F. (2018). Anapoles in free-standing III-V nanodisks enhancing second-harmonic generation. Nano Lett.

[22] Rocco D., Gili V. F., Ghirardini L. (2018). Tuning the second-harmonic generation in AlGaAs nanodimers via non-radiative state optimization [Invited]. Photonics Res.

[23] Yang Y., Kravchenko I. I., Briggs D. P., Valentine J. (2014). All-dielectric metasurface analogue of electromagnetically induced transparency. Nat. Commun..

[24] Shcherbakov M. R., Shorokhov A. S., Neshev D. N. (2015). Nonlinear interference and tailorable third-harmonic generation from dielectric oligomers. ACS Photonics.

[25] Hüttenhofer L., Tittl A., Kühner L., Cortés E., Maier S. A. (2021). Anapole-assisted absorption engineering in arrays of coupled amorphous gallium phosphide nanodisks. ACS Photonics.

[26] Mazzone V., Gongora J. S. T., Fratalocchi A. (2017). Near-field coupling and mode competition in multiple anapole systems. Appl. Sci..

[27] Huang T., Wang B., Zhang W., Zhao C. (2021). Ultracompact energy transfer in anapole-based metachains. Nano Lett..

[28] Zanganeh E., Song M., Valero A. C. (2021). Nonradiating sources for efficient wireless power transfer. Nanophotonics.

[29] Naureen S., Shahid N., Dev A., Anand S. (2013). Generation of substrate-free III-V nanodisks from user-defined multilayer nanopillar arrays for integration on Si. *Nanotechnology*.

[30] Marino G., Rocco D., Gigli C. (2021). Harmonic generation with multi-layer dielectric metasurfaces. Nanophotonics.

[31] Liu S., Keeler G. A., Reno J. L., Sinclair M. B., Brener I. (2016). III–V semiconductor nanoresonators—a new strategy for passive, active, and nonlinear all-dielectric metamaterials. Adv. Opt. Mater..

[32] Liu B., Hu M. L., Zhang Y. W. (2022). Strong near-field couplings of anapole modes and formation of higher-order electromagnetic modes in stacked all-dielectric nanodisks. *Chin. Phys. B*.

[33] Bulgakov E., Pichugin K., Sadreev A. (2021). Mie resonance engineering in two disks. Photonics.

[34] Lumerical Solutions FDTD. ..

[35] Polyanskiy M. Refractiveindex. Info, MediaWiki. ..

[36] Aspnes D. E., Kelso S. M., Logan R. A., Bhat R. (1986). Optical properties of Al x Ga 1− x as. J. Appl. Phys..

[37] Palik E. D. (1998). *Handbook of Optical Constants of Solids*.

[38] Fredriksson H., Alaverdyan Y., Dmitriev A. (2007). Hole-mask colloidal lithography. Adv. Mater..

[39] Yavas O., Svedendahl M., Quidant R. (2019). Unravelling the role of electric and magnetic dipoles in biosensing with Si nanoresonators. ACS Nano.

