# Supplementary material for: Manipulating light scattering and optical confinement in vertically stacked Mie resonators
Source: Nanophotonics. 2022 Nov 11;11(21):4755–64. doi: 10.1515/nanoph-2022-0605 (PMC11501431; doi:10.1515/nanoph-2022-0605)
Supplement: Supplementary file 1 — Supplementary Material Details [file j_nanoph-2022-0605_suppl.docx]

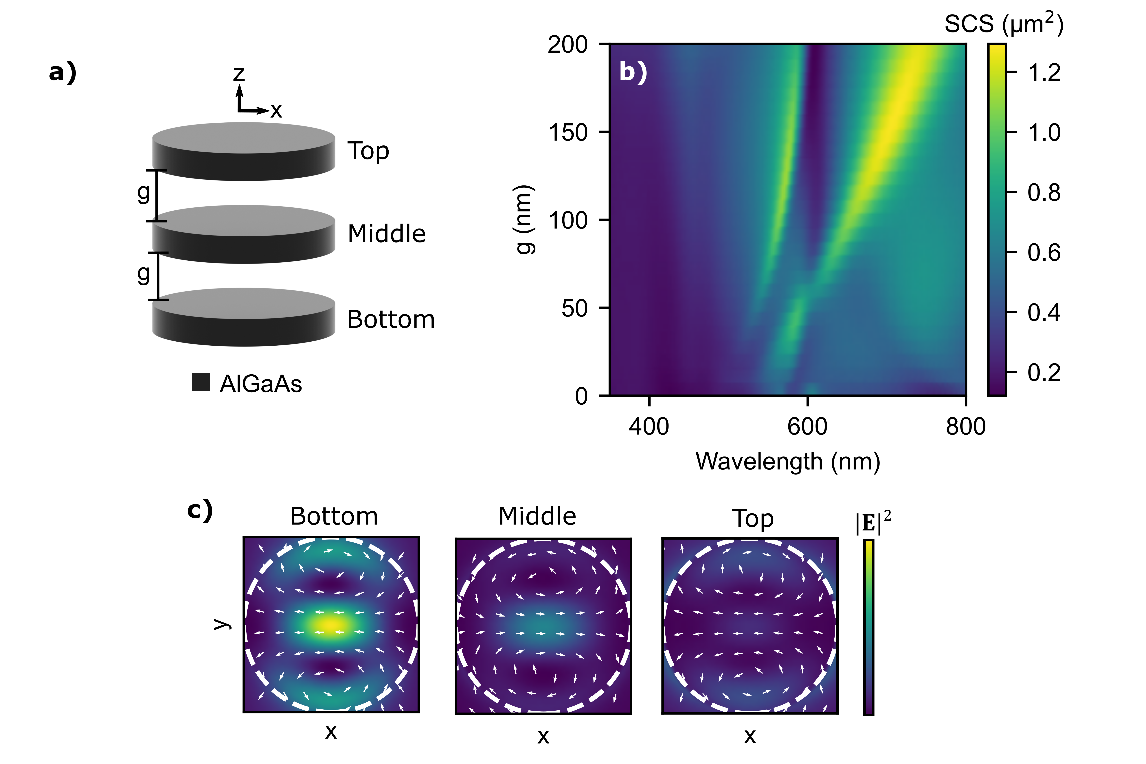


**SI 1.** Scattering cross section (SCS) of a stack of three disks. **a)** Schematic of the simulation setup. The light is incident in the –z direction and the air gap ‘g’ is the same between each disk. The disk all have a diameter of 350nm. **b)** The influence of ‘g’ on the SCS. The anapole state is around wavelength 610 nm and is invariant for ‘g’ above 70 nm. The resonances to either side red shifts considerably with increasing gap. **c)** Electric field distributions at a gap of 100 nm at the SCS dip position wavelength.


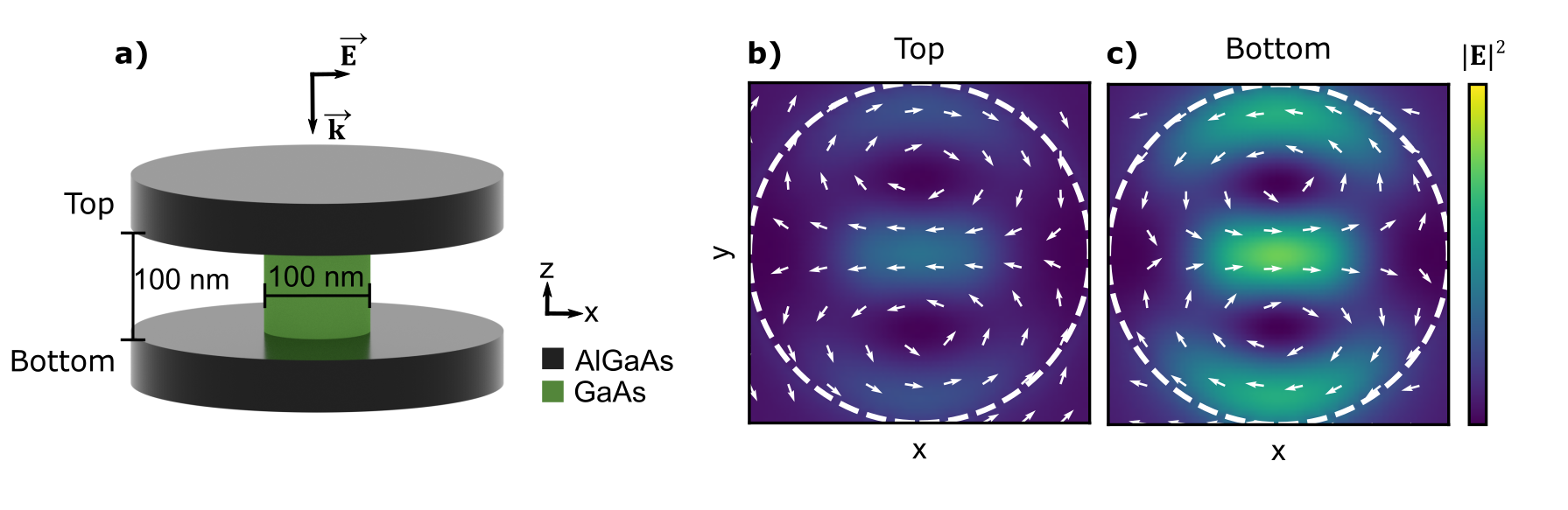


**SI 2.** Electric field intensities, |**E**|^2^, for each AlGaAs disk when they are connected by an GaAs stem. **a)** Schematic of the simulated structure. Light is incident in the –z direction and polarized along x. The gap between the disks is 100 nm and the diameter of the GaAs stem is 100 nm. **b),** **c)** The electric field intensities and orientation (white arrows) in the xy plane for the top and bottom disks. The fields are recorded at the middle z position of each disk. The fields and direction are typical for an anapole state.


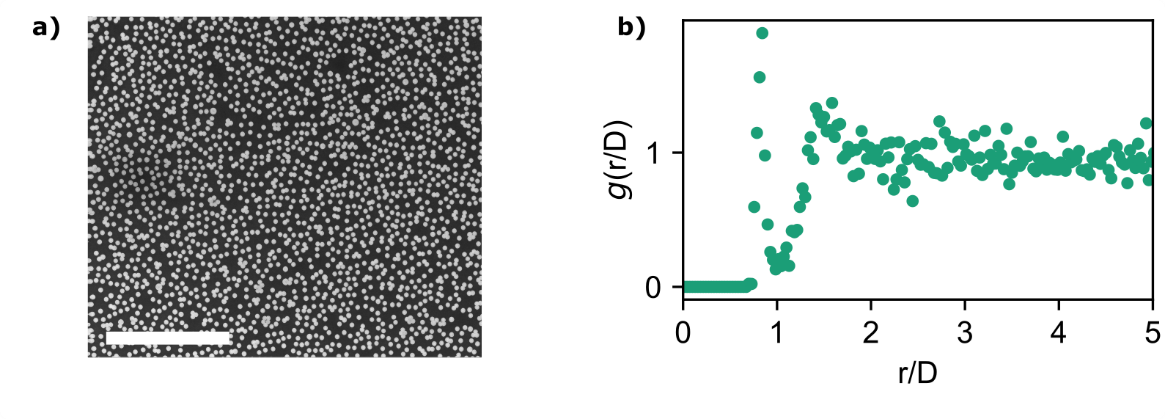


**SI 3.** The distribution of disks on the surface of the sample. **a)** Top view SEM image of finalized pillars, used to calculate the radial distribution function, $g(r/D)$. Scale bar is 10 µm.  **b)** Radial distribution function, $g(r/D)$, where $r$ is the distance from the center of a disk and $D$ is the diameter of the disk, in this case 350 nm. The peak around $r=D$ is due to clustering of particles, they are touching each other. Beyond that there is a small peak at one and a half to two diameters distance, signifying the closest neighbor distance for those that are not touching each other. $g(r/D)$ tends towards one beyond two diameters, showing absence of long range order.


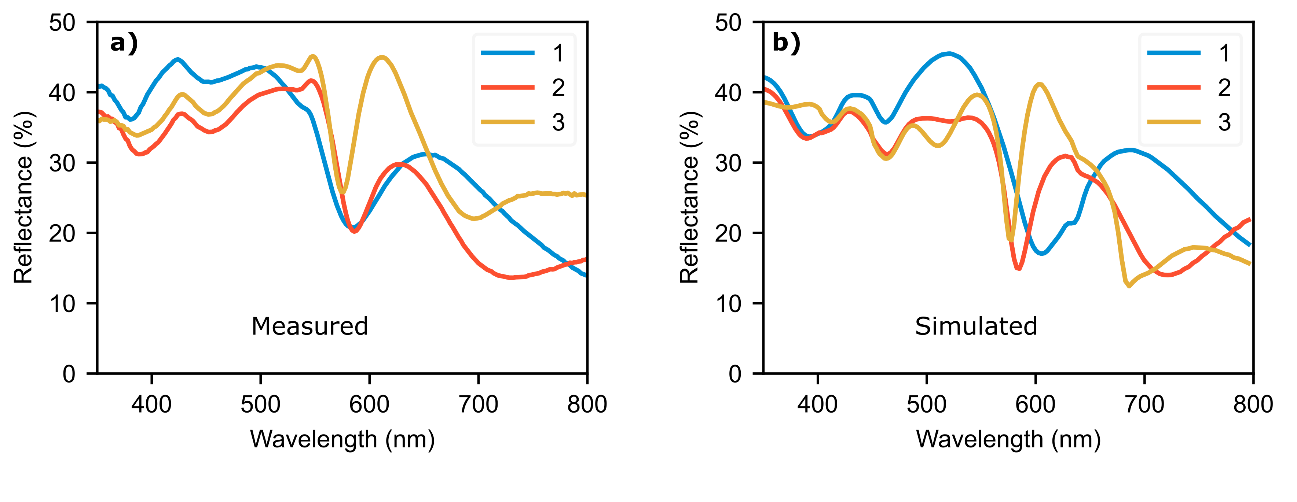


**SI 4.** Total reflectance from experiment **a)** and simulation **b)** for AlGaAs disks with diameter 350 nm and GaAs stems of about 50-70 nm diameter. The number in the legends signifies the number of disks in the stack. Note that in the one and two disk cases, the remaining multilayers are underneath the structure.


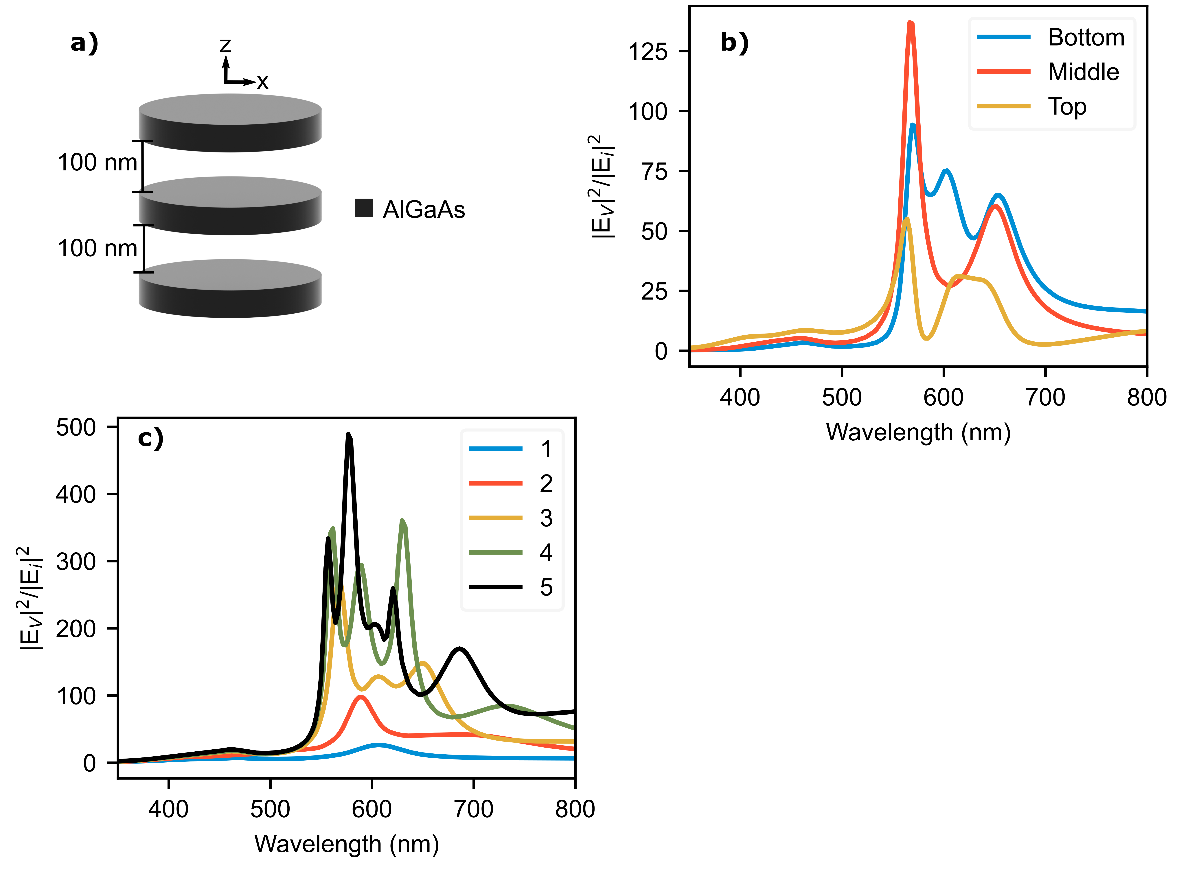


**SI 5.** Energy inside the disks ($\left| E_{v} \right|^{2}$) normalized to the incoming energy ($\left| E_{i} \right|^{2}$) for simulations of stacked disks in air. **a)** Schematic of the simulation setup, the light is incident in the –z direction. The disks are 350 nm in diameter and 50 nm thick. **b)** The energy inside each individual disks in the three disk configuration. Top is closest to the incoming radiation. **c)** Combined energies of stacked disks for each configuration. The numbers indicate how many disks are in the stack. E.g. the graph labelled 3 is the sum of all three graphs in **b)**.
